# Breast Cancer Risk and 6q22.33: Combined Results from Breast Cancer Association Consortium and Consortium of Investigators on Modifiers of *BRCA1*/*2*

**DOI:** 10.1371/journal.pone.0035706

**Published:** 2012-06-29

**Authors:** Tomas Kirchhoff, Mia M. Gaudet, Antonis C. Antoniou, Lesley McGuffog, Manjeet K. Humphreys, Alison M. Dunning, Stig E. Bojesen, Børge G. Nordestgaard, Henrik Flyger, Daehee Kang, Keun-Young Yoo, Dong-Young Noh, Sei-Hyun Ahn, Thilo Dork, Peter Schürmann, Johann H. Karstens, Peter Hillemanns, Fergus J. Couch, Janet Olson, Celine Vachon, Xianshu Wang, Angela Cox, Ian Brock, Graeme Elliott, Malcolm W.R. Reed, Barbara Burwinkel, Alfons Meindl, Hiltrud Brauch, Ute Hamann, Yon-Dschun Ko, GENICA Network, Annegien Broeks, Marjanka K. Schmidt, Laura J. Van ‘t Veer, Linde M. Braaf, Nichola Johnson, Olivia Fletcher, Lorna Gibson, Julian Peto, Clare Turnbull, Sheila Seal, Anthony Renwick, Nazneen Rahman, Pei-Ei Wu, Jyh-Cherng Yu, Chia-Ni Hsiung, Chen-Yang Shen, Melissa C. Southey, John L. Hopper, Fleur Hammet, Thijs Van Dorpe, Anne-Sophie Dieudonne, Sigrid Hatse, Diether Lambrechts, Irene L. Andrulis, Natalia Bogdanova, Natalia Antonenkova, Juri I. Rogov, Daria Prokofieva, Marina Bermisheva, Elza Khusnutdinova, Christi J. van Asperen, Robert A.E.M. Tollenaar, Maartje J. Hooning, Peter Devilee, Sara Margolin, Annika Lindblom, Roger L. Milne, José Ignacio Arias, M. Pilar Zamora, Javier Benítez, Gianluca Severi, Laura Baglietto, Graham G. Giles, kConFab, AOCS Study Group, Amanda B. Spurdle, Jonathan Beesley, Xiaoqing Chen, Helene Holland, Sue Healey, Shan Wang-Gohrke, Jenny Chang-Claude, Arto Mannermaa, Veli-Matti Kosma, Jaana Kauppinen, Vesa Kataja, Bjarni A. Agnarsson, Maria A. Caligo, Andrew K. Godwin, Heli Nevanlinna, Tuomas Heikkinen, Zachary Fredericksen, Noralane Lindor, Katherine L. Nathanson, Susan M. Domchek, SWE-BRCA, Niklas Loman, Per Karlsson, Marie Stenmark Askmalm, Beatrice Melin, Anna von Wachenfeldt, HEBON, Frans B. L. Hogervorst, Martijn Verheus, Matti A. Rookus, Caroline Seynaeve, Rogier A. Oldenburg, Marjolijn J. Ligtenberg, Margreet G.E.M. Ausems, Cora M. Aalfs, Hans J.P. Gille, Juul T. Wijnen, Encarna B. Gómez García, EMBRACE, Susan Peock, Margaret Cook, Clare T. Oliver, Debra Frost, Craig Luccarini, Gabriella Pichert, Rosemarie Davidson, Carol Chu, Diana Eccles, Kai-Ren Ong, Jackie Cook, Fiona Douglas, Shirley Hodgson, D. Gareth Evans, Rosalind Eeles, Bert Gold, Paul D.P. Pharoah, Kenneth Offit, Georgia Chenevix-Trench, Douglas F. Easton, on behalf of BCAC/CIMBA

**Affiliations:** 1 Memorial Sloan-Kettering Cancer Center (MSKCC): Clinical Genetics Service, Memorial Sloan-Kettering Cancer Center, New York, New York, United States of America (TK, MG, KO); Department of Environmental Medicine, New York University Cancer Institute, New York University, New York, New York, United States of America (TK), American Cancer Society, Atlanta, Georgia, United States of America (MG); 2 Studies of Epidemiology and Risk Factors in Cancer Heredity (SEARCH): Department of Oncology and Department of Public Health and Primary Care, University of Cambridge, Cambridge, United Kingdom (ACA, LM, MKH, AMD, PDPP, DFE); 3 The Copenhagen Breast Cancer Study and The Copenhagen General Population Study (CGPS): Department of Clinical Biochemistry (SEB, BGN); Department of Breast Surgery, Herlev Hospital (HF), Copenhagen University Hospital, University of Copenhagen, Denmark; 4 Seoul Breast Cancer Study (SEBCS): Seoul National University College of Medicine and National Cancer Center, Seoul, Korea; Department of Surgery, Ulsan University College of Medicine, Ulsan, Korea (DK, KYY, DYN, SHA); 5 Hannover Breast Cancer Study (HABCS): Clinics of Obstetrics and Gynecology and Clinic of Radiation Oncology, Hannover Medical School, Hannover, Germany (TD, PS, JHK, PH); 6 Mayo Clinic Breast Cancer Study (MCBCS): Mayo Clinic, Rochester, Minnesota, United States of America (FJC, JO, CV, XW); 7 Sheffield Breast Cancer Study (SBCS): Department of Oncology, Sheffield University Medical School, Sheffield, United Kingdom (AC, IB, GE, MWRR); 8 German Consortium for Hereditary Breast and Ovarian Cancer (GC-HBOC): Institute of Human Genetics, German Cancer Research Center, Heidelberg, Germany (BB); Department of Obstetrics and Gynecology, Division of Tumor Genetics, Technical University of Munich, Munich, Germany (AM); 9 Gene Environment Interaction and Breast Cancer in Germany (GENICA): Dr. Margarete Fischer-Bosch-Institute of Clinical Pharmacology, Stuttgart, and University Tübingen, Stuttgart and Tübingen, Germany (HB, Christina Justenhoven); Molecular Genetics of Breast Cancer, Deutsches Krebsforschungszentrum, Heidelberg, Germany (UH); Department of Internal Medicine, Evangelische Kliniken Bonn gGmbH, Johanniter Krankenhaus, Bonn, Germany (YDK,); Institute of Pathology, Medical Faculty of the University of Bonn, Bonn, Germany (Hans-Peter Fischer); Institute for Prevention and Occupational Medicine of the German Social Accident Insurance, Bochum, Germany (Thomas Brüning, Beate Pesch, Volker Harth, Sylvia Rabstein); 10 Amsterdam Breast Cancer Study (ABCS): Netherlands Cancer Institute, Departments of Experimental Therapy, Epidemiology and Molecular Pathology, Amsterdam, The Netherlands (AB, MKS, LJVV, LMB); 11 British Breast Cancer Study (BBCS): Breakthrough Breast Cancer Research Centre, The Institute of Cancer Research, London, United Kingdom (NJ, OF); Department of Epidemiology and Population Health, London School of Hygiene and Tropical Medicine, London, United Kingdom (LG, JP); 12 ICR Familial Breast Cancer Study (FBCS): Section of Cancer Genetics, Institute of Cancer Research, Sutton, Surrey, United Kingdom (CT, SS, AR, NR); 13 Taiwanese Breast Cancer Study (TWBCS): Institute of Biomedical Sciences, Academia Sinica, Taipei, Taiwan (CNH, CYS); Taiwan Biobank, Academia Sinica, Taiwan (PEW); Departments of Surgery, Tri-Service General Hospital, Taipei, Taiwan (JCY); 14 Australian Breast Cancer Family Study (ABCFS): Genetic Epidemiology Laboratory, Department of Pathology (MCS, FH), Centre for Molecular Environmental Genetic and Analytic Epidemiology (JLH), The University of Melbourne, Victoria, Australia; 15 Leuven Multidisciplinary Breast Centre (LMBC): Katholieke Universiteit Leuven–Multidisciplinary Breast Clinic (TVD, ASD, SH), Vesalius Research Center (DL), Leuven, Belgium; 16 Ontario Familial Breast Cancer Registry (OFBCR): Ontario Cancer Genetics Network, Cancer Care Ontario, Ontario, Canada; Fred A. Litwin Center for Cancer Genetics, Samuel Lunenfeld Research Institute, Mount Sinai Hospital, Ontario, Canada; Department of Molecular Genetics, University of Toronto, Toronto, Ontario, Canada (ILA); 17 Hannover-Minsk Breast Cancer Study (HMBCS): N.N. Alexandrov Research Institute of Oncology and Medical Radiology, Minsk, Belarus (NB, NA, JIR); 18 Hannover-Ufa Breast Cancer Study (HUBCS): Institute of Biochemistry and Genetics, Ufa Scientific Center of Russian Academy of Sciences, Ufa, Russia (DP, MB, EK); 19 Leiden University Medical Center Breast Cancer Study (ORIGO): Department of Medical Oncology, Erasmus Medical Center, Rotterdam, The Netherlands (MJH); Departments of Surgical Oncology (RAEMT), Clinical Genetics (CJVA), Human Genetics and Pathology (PD), Leiden University Medical Center, Leiden, The Netherlands; 20 Karolinska Breast Cancer Study (KARBAC): Department of Molecular Medicine and Surgery (AL) and Department of Oncology-Pathology, Karolinska Institutet and Karolinska University, Stockholm, Sweden (SM); 21 Spanish National Cancer Center Breast Cancer Study (CNIO-BCS): Genetic and Molecular Epidemiology Group (RLM) and Human Genetics Group (JB), Spanish National Cancer Centre, Madrid, Spain; Centro de Investigación Biomédica en Red de Enfermedades Raras, Valencia, Spain (JB); Monte Naranco Hospital, Oviedo, Spain (JIA); La Paz University Hospital, Madrid, Spain (MPZ); 22 Melbourne Collaborative Cohort Study (MCCS): Cancer Epidemiology Centre, The Cancer Council, Victoria, Melbourne, Australia and Centre for Molecular Environmental, Genetic, and Analytic Epidemiology, School of Population Health, The University of Melbourne, Australia (GGG, MCS, GS, LB); 23 Kathleen Cuningham Foundation Consortium for Research into Familial Breast Cancer (kConFaB): Queensland Institute of Medical Research, Brisbane, and the Australian Ovarian Cancer Study Group, Peter MacCallum Cancer Center, Melbourne, Australia (ABS, JB, XC, HH, SH, GCT); 24 Genetic Epidemiology Study of Breast Cancer by Age 50 (GESBC): Division of Cancer Epidemiology, German Cancer Research Center [DFKZ], Heidelberg, Germany (JC-C), and Molecular Biology Laboratory, Department of Gynecology and Obstetrics, University of Ulm, Ulm, Germany (SW-G); 25 Kuopio Breast Cancer Project (KBCP): Institute of Clinical Medicine, Department of Pathology, University of Eastern Finland, and Kuopio University Hospital, Biocenter Kuopio, Kuopio Finland (AM, VMK, JK); Department of Oncology, Vaasa Central Hospital, Vaasa, Finland, and Department of Oncology, Kuopio University Hospital, Kuopio, Finland (VK); 26 Iceland Landspitali–University Hospital (ILUH): Department of Pathology, Landspitali-University Hospital and University of Iceland School of Medicine, Reykjavik, Iceland (BAA); 27 Division of Surgical, Molecular and Ultrastructural Pathology, Department of Oncology University of Pisa and Pisa University Hospital, Pisa, Italy (MAC); 28 Fox Chase Cancer Center (FCCC): Fox Chase Cancer Center, Philadelphia, Pennsylvania, United States of America (AKG); 29 Helsinki Breast Cancer Study (HEBCS): Department of Obstetrics and Gynecology, Helsinki University Central Hospital, Helsinki, Finland (HN, TH); 30 Mayo Clinic Familial Breast and Ovarian Cancer Study (MAYO), Mayo Clinic, Rochester, Minnesota, United States of America (FJC, ZF, NL); 31 The University of Pennsylvania (UPENN): Department of Medicine and Abramson Cancer Center, University of Pennsylvania, Philadelphia, Pennsylvania, United States of America (KLN, SMD); 32 The Swedish BRCA1 and BRCA2 study collaborators (SWE-BRCA): Department of Oncology, Lund University Hospital, Lund, Sweden (NL); Department of Oncology, Sahlgrenska University Hospital, Gothenburg, Sweden (PK); Department of Oncology, University Hospital, Linköping, Sweden (MSA); Department of Radiation Sciences, Oncology, Umeå University, Umeå Sweden (BM); Department of Oncology, Karolinska University Hospital, Stockholm, Sweden (AVW); 33 The Hereditary Breast and Ovarian Cancer Research Group Netherlands (HEBON): Coordinating Center: Netherlands Cancer Institute, Amsterdam, The Netherlands (FBLH, MV, MAR); Erasmus Medical Center, Rotterdam, The Netherlands (CS, RAO); Leiden University Medical Center, Leiden, The Netherlands (JTW); Radboud University Nijmegen Medical Center, Nijmegen, The Netherlands (MJL); University Medical Center Utrecht, Utrecht, The Netherlands (MGA); Amsterdam Medical Center, Amsterdam, The Netherlands (CMA); Vrije Universiteit University Medical Center, Amsterdam, The Netherlands (HJPG); University Hospital Maastricht, Maastricht, The Netherlands (EBG); 34 Epidemiological study of BRCA1 and BRCA2 mutation carriers (EMBRACE): Centre for Cancer Genetic Epidemiology, Department of Public Health and Primary Care, University of Cambridge, Cambridge, United Kingdom (SP, MC, CTO, DF, CL); Genetic Medicine, Manchester Academic Health Sciences Centre, Central Manchester University Hospitals National Health Service (NHS) Foundation Trust, Manchester, United Kingdom (DGE); Oncogenetics Team, The Institute of Cancer Research and Royal Marsden NHS Foundation Trust, London, United Kingdom (RE); Clinical Genetics, Guy’s and St. Thomas’ NHS Foundation Trust, London, United Kingdom (GP); Ferguson-Smith Centre for Clinical Genetics, Yorkhill Hospitals, Glasgow, United Kingdom (RD); Yorkshire Regional Genetics Service, Leeds, United Kingdom (CC); Wessex Clinical Genetics Service, Princess Anne Hospital, Southampton, United Kingdom (DE); West Midlands Regional Genetics Service, Birmingham Women’s Hospital Healthcare NHS Trust, Edgbaston, Birmingham, United Kingdom (KRO); Sheffield Clinical Genetics Service, Sheffield Children’s Hospital, Sheffield, United Kingdom (JC); Institute of Human Genetics, Centre for Life, Newcastle Upon Tyne Hospitals NHS Trust, Newcastle Upon Tyne, United Kingdom (FD); Clinical Genetics Department, St Georges Hospital, University of London, London, United Kingdom (SH); 35 National Cancer Institute (NCI): Clinical Genetics Branch, National Cancer Institute, Rockville, Maryland, United States of America (BG); National Cancer Institute, National Institutes of Health, United States of America

## Abstract

Recently, a locus on chromosome 6q22.33 (rs2180341) was reported to be associated with increased breast cancer risk in the Ashkenazi Jewish (AJ) population, and this association was also observed in populations of non-AJ European ancestry. In the present study, we performed a large replication analysis of rs2180341 using data from 31,428 invasive breast cancer cases and 34,700 controls collected from 25 studies in the Breast Cancer Association Consortium (BCAC). In addition, we evaluated whether rs2180341 modifies breast cancer risk in 3,361 *BRCA1* and 2,020 *BRCA2* carriers from 11 centers in the Consortium of Investigators of Modifiers of *BRCA1/2* (CIMBA). Based on the BCAC data from women of European ancestry, we found evidence for a weak association with breast cancer risk for rs2180341 (per-allele odds ratio (OR) = 1.03, 95% CI 1.00–1.06, p = 0.023). There was evidence for heterogeneity in the ORs among studies (I^2^ = 49.3%; p = <0.004). In CIMBA, we observed an inverse association with the minor allele of rs2180341 and breast cancer risk in *BRCA1* mutation carriers (per-allele OR = 0.89, 95%CI 0.80–1.00, p = 0.048), indicating a potential protective effect of this allele. These data suggest that that 6q22.33 confers a weak effect on breast cancer risk.

## Introduction

Genome-wide association analyses have recently identified multiple loci conferring genetic susceptibility to breast cancer [1], [2], [3], [4]. Due to the low relative risks associated with such loci, however, very large case-control studies are required to confirm these and estimate the associated risks reliably [5], [6].

Recently, a putative breast cancer susceptibility locus at chromosome 6q22.33 (tagged by rs2180341) was identified by a two-stage genome wide-association study (GWAS) based on a phase 1 analysis of 299 Ashkenazi Jewish (AJ) controls and 249 AJ kindreds with family history of breast cancer and no known *BRCA* mutation, followed by phase 2 analysis of 979 AJ controls and 950 AJ breast cancer cases [7]. The association signal spanned an approximately 100 kb region with two candidate genes, *ECHDC1* and *RNF146*, mapping to this locus. In a follow-up study, an association was observed in an independent analysis of 1,953 breast cancer cases and 1,467 controls of non-AJ, predominantly European ancestry (per-allele OR 1.18, 95% CI 1.04–1.33, p = 0.0083) with some evidence of a stronger association for ER+ than ER- tumors [8].

Our objective in the current analysis was to further investigate the association of the 6q22.33 locus with breast cancer risk. To this end, we genotyped rs2180341 in 27,950 invasive breast cancer cases and 32,219 controls from 23 case-control studies of primarily European ancestry and 2 studies of Asians included in the Breast Cancer Association Consortium (BCAC). We also evaluated whether rs2180341 was associated with breast cancer risk in *BRCA1* or *BRCA2* mutation carriers, by genotyping 5,381 mutation carriers from 11 studies in the Consortium of Investigators on Modifiers of *BRCA1*/*2* (CIMBA).

**Figure 1:**
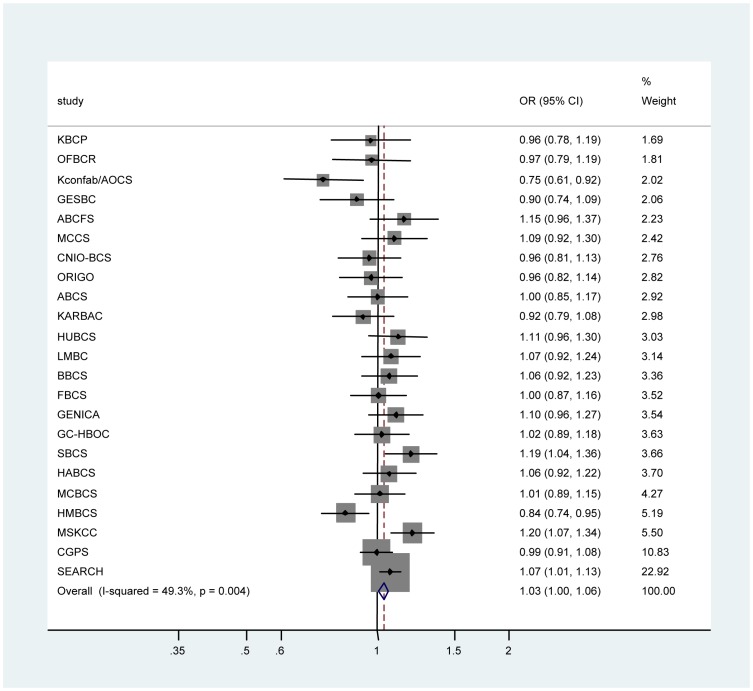
Forest plot of SNP rs2180341 per-allele odds ratios (ORs) and 95% confidence intervals (CIs) with the risk of breast cancer among studies from Breast Cancer Association Consortium (BCAC) breast cancer cases and controls of European ancestry. Studies are weighted and ranked according to the inverse of the between-study and within study variation of the log odds ratio, which is also represented by the size of the shaded box around the study-specific point estimate. The solid line indicates the OR = 1 and the dashed lined indicates the summary OR of all studies. A description of the study acronyms can be found in the Supporting Information S1.

**Table 1:** Summary odds ratios^1^ (ORs) and 95% confidence intervals (CIs), adjusted for age and study, for SNP rs2180341 genotypes and breast cancer risk, Breast Cancer Association Consortium (BCAC).

Genotype	No. of studies	No. of cases	No. of controls	MAF^2^	Pooled				
					OR^1^	(95% CI)			p-value
**Among Women of European Ancestry**									
*AA*		15,526	18,154		1				
*AG*		10,644	12,142		1.03	(0.99	–	1.06)	0.13
*GG*	23	1,780	1,923	24.8	1.07	(1.00	–	1.15)	0.044
*recessive*					1.06	(0.99	–	1.14)	0.082
*per allele*					1.03	(1.00	–	1.06)	0.023
									
**Among Women of Asian Ancestry** 3									
*AA*		1,604	1,228		1				
*AG*		1,041	785		0.99	(0.87	–	1.12)	0.85
*GG*	2	191	136	24.6	0.99	(0.78	–	1.25)	0.93
*recessive*					0.99	(0.79	–	1.25)	0.96
*per allele*					0.99	(0.90	–	1.09)	0.85

1 ORs were adjusted for study.

2 MAF = minor allele frequency.

3 Ten studies contributed samples from women self-described as Asian. Two of these studies were conducted in Asian countries and contributed the majority of Asian samples.

## Materials and Methods

### Ethics Statement

Ethics committee approval was obtained for the collection and genetic analysis of all samples, and an informed written consent was obtained from all participants. For detailed description, see Supporting Information S1.

### Breast Cancer Association Consortium (BCAC)

Twenty-five case-control studies (described in Supporting Information S1) contributed data to these analyses. Data were available on age at study recruitment and ethnicity. Studies were conducted in Europe, North America, and Australia, among women of primarily European descent, and in Southeast Asia. For one study (MSKCC, see study acronyms in Supporting Information S1), we included previously genotyped data from a follow-up analysis reported recently [8]. These data represent an independent group of breast cancer cases and controls of non-AJ European ancestry not used in the prior two-stage GWAS in AJ population [7].

In the current dataset, we excluded breast cancer cases with *in situ* diagnoses (736 cases). Final analyses included 27,950 invasive breast cancer cases and 32,219 controls of European ancestry, as well as 2,836 invasive breast cancer cases and 2,149 controls of Asian ancestry. All studies received approval from their institutional review committees and participants provided informed consent or were analyzed under specific coding procedures (ABCS).

### Consortium of Modifiers of BRCA1 and BRCA2 (CIMBA)

Eleven studies (described in Supporting Information S1) from Europe, North America, and Australia contributed samples from carriers to these analyses. Eligible female carriers were aged 18 years or older and had pathogenic mutations in *BRCA1* and/or *BRCA2*. Data were available on year of birth, age at study recruitment, age at cancer diagnosis, age of bilateral prophylactic mastectomy, *BRCA1* and *BRCA2* mutation description, and ethnicity. Final analyses included 2,776 invasive breast cancer cases and 2,605 unaffected mutation carriers.

**Table 2:** Study-adjusted association between SNP rs2180341 and breast cancer risk by age among cases and controls of European ancestry, Breast Cancer Association Consortium (BCAC).

Genotype	N cases	N controls	OR	(95% CI)			N cases	N controls	OR	(95% CI)			N cases	N controls	OR	(95% CI)			N cases	N controls	OR	(95% CI)				p for interaction
	Age <40 years						Age 40–49 years						Age 50–59 years						Age ≥60 years							
*AA*	1,917	2,271	1				4,327	3,317	1				4,700	5,005	1				4,421	5,467	1					
*AG*	1,272	1,575	0.98	(0.88	–	1.10)	2,932	2,251	0.99	(0.92	–	1.08)	3,126	3,316	1.01	(0.94	–	1.08)	3,197	3,616	1.07	(1.00	–	1.14)		
*GG*	193	251	0.94	(0.75	–	1.19)	498	358	1.02	(0.86	–	1.21)	556	520	1.21	(1.05	–	1.39)	516	556	1.08	(0.94	–	1.24)		
*recessive*			0.95	(0.76	–	1.19)			1.02	(0.87	–	1.20)			1.21	(1.06	–	1.38)			1.05	(0.92	–	1.21)		
*per allele*			0.98	(0.90	–	1.07)			1	(0.94	–	1.07)			1.05	(1.00	–	1.11)			1.05	(1.00	–	1.11)		0.044

### Genotyping

For most of the BCAC part of the study, the genotyping of rs2180341 was performed by TaqMan allelic discrimination assay using the standard protocol, described previously [8]. For the genotyping of 3 BCAC centers (see Supporting Information S1) and all CIMBA studies, the Sequenom platform was used (Sequenom, San Diego, CA, USA). Briefly, the matrix-assisted laser desorption/ionization time of flight mass spectrometry (MALDI-TOF MS) was used to determine allele-specific primer extension products using Sequenom’s MassARRAY system and iPLEX technology (Sequenom, San Diego, CA, USA). The design of oligonucleotides was carried out according to the guidelines of Sequenom and performed using MassARRAY Assay Design software (version 3.1). Robust quality control criteria, established by BCAC/CIMBA, were applied as detailed in previous consortium studies [9], [10], [11]. Briefly, the genotyping concordance was verified with internal duplicates and overall data quality was ensured using independent genotyping of CEU samples by each genotyping center. We excluded all samples that failed on two or more of the SNPs genotyped in a particular BCAC/CIMBA genotyping round. All studies met the specified criteria for call rate (>95%), and Hardy-Weinberg Equilibrium (HWE; p≥0.001).

**Table 3:** Association between SNP rs2180341 and breast cancer risk by estrogen receptor (ER) status among cases and controls of European ancestry, Breast Cancer Association Consortium (BCAC).

Genotype	No. of cases	No. of controls	OR^1^	(95% CI)			No. of cases	No. of controls	OR^1^	(95% CI)			p for tumor heterogeneity
	ER+						ER-						
AA	6,584	19,554	1				1,930	19,554	1				
AG	4,632	13,067	1.04	(1.00	−	1.09)	1,309	13,067	1.01	(0.93	–	1.10)	
GG	740	2,079	1.07	(0.97	−	1.18)	186	2,079	0.94	(0.79	−	1.12)	
recessive			1.05	(0.96	−	1.16)			0.93	(0.79	−	1.11)	
per allele			1.04	(1.00	−	1.08)			0.99	(0.93	−	1.06)	0.21

### Statistical Analyses for BCAC

Study-adjusted, fixed-effects models, weighted for each study by the within- and between-study variances, were used to estimate pooled odds ratios (OR) and 95% confidence intervals (CI). The percent of between-study heterogeneity was estimated using the I^2^ statistic [12], [13]. ORs for rs2180341 were estimated under the log-additive model (per-allele OR), the recessive model, and the 2 degree of freedom (2 df) model, with the common homozygote as a reference category. Women of non-European ancestry in studies of predominantly European ancestry were excluded from the analysis. Separate estimates for women of Asian ancestry were performed. Analyses stratified on age and estrogen receptor (ER) status among cases were also performed; missing data for each variable were excluded from the respective analyses. The p-values for interaction with age were calculated by comparing the log likelihood estimates of models with and without an interaction term for age and genotype (each coded as an ordinal categorical variable). The p-value for tumor heterogeneity by ER status was based on the comparison of ORs for the ER-positive (ER+) and ER-negative (ER-) tumors.

### Statistical Methods for CIMBA

Hazard ratios (HRs) and 95% CIs were estimated using a weighted Cox regression approach as described in detail elsewhere [14]. Briefly, to correct for potential bias due to over-sampling of affected carriers the affected and unaffected mutation carriers were differentially weighted such that the observed breast cancer incidences in the mutation carrier dataset agreed with external breast cancer incidences for *BRCA1* and *BRCA2* mutation carriers. We used a robust variance approach to allow for the dependence among related mutation carriers. We also adjusted for study, country, ethnicity, and year of birth. Relative risk estimates were calculated separately for *BRCA1* and *BRCA2* mutation carriers.

Mutation carriers were censored at the first breast or ovarian cancer or bilateral prophylactic mastectomy. Carriers who developed either cancer were censored at the time of bilateral prophylactic mastectomy only if it occurred more than a year prior to the cancer diagnosis (to avoid censoring at bilateral mastectomies related to diagnosis in which rounded ages were used). The remaining carriers were censored at the age of last observation. This was defined either by the age at interview or age at follow-up depending on the information provided by the participating center. Carriers censored at diagnosis of breast cancer were considered affected in the analysis. Carriers with a censoring/last follow-up age older than age 80 were censored at age 80 because there are no reliable cancer incidence rates for *BRCA1/2* mutation carriers beyond age 80.

All analyses were performed with STATA (Version 10.0).

**Table 4:** Adjusted^1^, weighted hazard ratios (HRs) and 95% confidence intervals (CIs) for the association between SNP rs2180341 genotype and breast cancer risk, in the Consortium of Investigators of Modifiers of BRCA1/2 (CIMBA).^2^

Genotype	No. of Studies	N affected	N unaffected	MAF^3^	HR	(95% CI)			p-value
**Among ** ***BRCA1*** ** Mutation Carriers**									
AA		1,041	934		1				
AG		582	602		0.87	(0.76	–	1.00)	0.05
GG	11	96	106	24.8	0.85	(0.64	–	1.11)	0.23
recessive					0.89	(0.68	–	1.16)	0.4
per allele					0.89	(0.80	–	1.00)	0.048
**Among ** ***BRCA2*** ** Mutation Carriers**									
AA		605	528		1				
AG		384	384		0.97	(0.82	–	1.15)	0.73
GG	11	68	51	25.2	1.15	(0.84	–	1.56)	0.38
recessive					1.14	(0.86	–	1.56)	0.33
per allele					1.02	(0.90	–	1.16)	0.75

1 Adjusted for birth year and study.

2 Restricted to women of European descent.

3 MAF = Minor allele frequency.

**Figure 2:**
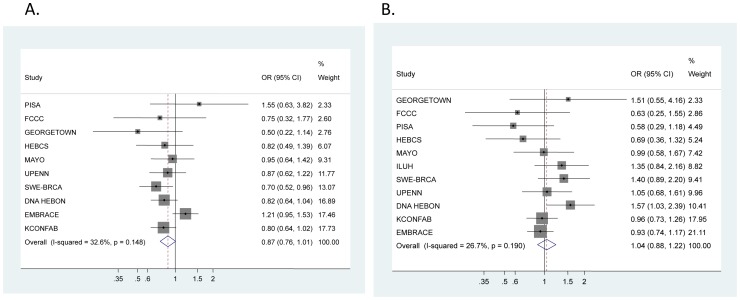
SNP rs2180341 per-allele hazard ratios (HRs) and 95% confidence intervals (CIs) among Consortium of Investigators of Modifiers of *BRCA1/2* (CIMBA) in A. *BRCA1* mutation carriers B. *BRCA2* mutation carriers. Studies are weighted and ranked according to the inverse of the between-study and within study variation of the log odds ratio, which is also represented by the size of the shaded box around the study-specific point estimate. The solid line indicates the OR = 1 and the dashed lined indicates the summary OR of all studies. A description of the study acronyms can be found in Supporting Information S1.

## Results

### Description of BCAC Study Population

The mean (±SD) age was 53.1 (±13.1) years for invasive cases, 52.7 (±11.8) years for controls. A total of 88.9% of invasive cases and 92.9% of controls were of European-descent. Other women were of Asian ancestry (9.0% cases and 6.2% controls) or unknown ancestry (2.1% cases and 0.9% controls, respectively).

Among controls of European descent, the minor allele frequency (MAF) of rs2180341 ranged from 22.6% to 28.7% (mean 24.8%; Supporting Information S1). The MAF was similar for controls of Asian descent (24.5%, mean of 2 studies).

### Association Between rs2180341 and Risk of Breast Cancer

There was some evidence for an association between the G allele and breast cancer risk (per-allele OR 1.03, 95% CI 1.00–1.06, p = 0.023, Table 1). The highest risk was observed for GG homozygotes (OR = 1.07, 95% CI 1.00–1.15; p = 0.044). Significant between-study heterogeneity (Figure 1) was observed for the per-allele ORs for women of European ancestry (I^2^ = 49.3%; p = 0.004), which was mainly attributable to the strong inverse associations for kConFab/AOCS and HMBCS, and a strong positive association for MSKCC and SBCS. Exclusion of these studies did not alter the overall magnitude of the relative risk estimate and there was no longer evidence of between-study heterogeneity (per-allele OR = 1.03, 95% CI 1.00–1.06, p = 0.034; between-study heterogeneity: I^2^ = 0.0%, p = 0.80).

There was an indication of effect modification by age (p for interaction = 0.044; Table 2); we observed no association in the <40 or 40–49 year age groups, but increased association in the age 50–59 and >60 year age groups (OR = 1.05, 95% CI 1.00–1.11 and OR = 1.05, 95% CI 1.00–1.11, respectively). The association in the age group of 50–59 was stronger under the recessive model of analysis (OR = 1.21, 95% CI 1.06–1.38, p = 0.006).

Among women of Asian ancestry, we did not observe an association with overall breast cancer risk and the 6q22.33 locus (Table 1), but there was significant between-study heterogeneity (I^2^ = 89.3%; p = 0.002).

We examined the association of rs2180341 with breast cancer by ER status, which was available from 19 studies that were conducted predominantly among women of European ancestry. There was no significant difference in the per-allele OR for rs2180341 in the risk of ER+ and ER- disease (p for tumor heterogeneity = 0.21; Table 3). However, the per-allele OR estimate for ER+ tumors (OR = 1.04, 95% CI 1.00–1.08) was greater than that for ER- tumors (OR = 0.99, 95% CI 0.93–1.06).

### Description of CIMBA Study Population

We also examined the association with rs2180341 in women with *BRCA1* or *BRCA2* mutations based on data from 2,776 invasive breast cancer cases and 2,605 unaffected carriers. Sixty-two percent of the carriers had a *BRCA1* mutation. On average, *BRCA1* affected carriers were censored at age 41.0 (±9.3 SD), *BRCA1* unaffected carriers at age 43.1 (±12.7 SD), *BRCA2* affected carriers at age 44.3 (±13.5 SD), and *BRCA2* unaffected carriers at age 45.1 (±13.5 SD). All study subjects were of European-descent. Genotype frequencies were in HWE for all studies. Among unaffected carriers, the MAF of rs2180341 ranged from 19.2% to 33.3% (see Supporting Information S1) with a mean of 25.0% (Table 4).

### Association Between rs2180341 and Breast Cancer Risk in BRCA1/2 Carriers

The minor allele was statistically significantly associated with a lower breast cancer risk for *BRCA1* mutation carriers, (per-allele HR = 0.89, 95% CI 0.80–1.00, p = 0.04, Table 4, Figure 2A). The per-allele HR estimate for *BRCA2* mutation carriers was 1.02, 95% 0.90–1.16, p = 0.75 (Table 4, Figure 2B). There was no evidence of between-study heterogeneity for the estimates among *BRCA1* (p = 0.15) or *BRCA2* (p = 0.19) mutation carriers (see forest plots in Figure 2). ER status was not available for the affected mutation carriers at the time of analysis.

## Discussion

While several recent studies on different ancestries reported an association of 6q22.33 with breast cancer risk [15], [16], none of the prior breast cancer GWAS of European populations have independently identified the 6q22.33 region, possibly due to limited power in the first stage design of these studies. Noting that the true magnitude of the effect for 6q22.33 on overall breast cancer risk is likely to be small, in this report we sought to provide a more precise estimate of breast cancer risk associated with the 6q22.33 locus in a study of more than 50,000 breast cancer cases and controls ascertained through the international BCAC. After restricting the analysis to women of European ancestry, the overall estimate showed a weak, per-allele association (OR = 1.03, Table 1, Figure 1), which is smaller than the first replication analysis in non-AJ European populations (per-allele OR = 1.19) [8]. Findings from the BCAC study are also consistent with previous observations that a higher OR was found for minor allele homozygotes. Notably, there was significant between-study heterogeneity in the BCAC data, even when the analysis was limited to women of European ancestry (p = 0.004, Figure 1). For example, while some centers, (MSKCC, SBCS, or SEARCH) showed comparable effect size with original observations from AJ GWAS, other centers (e.g. kConFab, GESBC, HMBCS) yielded risk estimates in the opposite direction. Such “flip-flop” associations may be due to chance, but have also been observed in the setting of other known associations, and may result from local differences in linkage disequilibrium structure between selected populations, even within the same ethnicities [17]. Moreover, for two centers ascertained from the UK population (SBCS and SEARCH) representing a large portion of the BCAC data (n = 15,478), the magnitude of the association was more comparable to prior observations in AJ as well as European ancestry; a per allele OR = 1.09 (95% CI 1.03–1.15; p = 0.002) was noted in the combined SBCS and SEARCH study populations compared to OR = 1.18 (95%CI 1.04–1.35, p = 0.008) in a U.S. study of non-Ashkenazi Caucasians [8]. We hypothesize that the heterogeneity between studies may largely be attributed to local population stratification; for example OR estimates observed in the UK studies differed from those in the Copenhagen (CGPS) study. While ancestry-informative panels or principal components from genome-wide scans will need to be incorporated into the present meta-analysis to quantify potential population stratification, such markers were unavailable for the current study. With the completion of a large ongoing consortia effort on breast cancer susceptibility (ICOGs) however, this information will be readily accessible to test the possible confounding effect of the population substructure on the observed association.

The chance is also a likely explanation of observed heterogeneity because individual estimates based on studies with wider 95% confidence intervals, such as kConFab or GESBC, may be more susceptible to random error [18]. Excluding “outlier” studies from the present analysis did not alter the magnitude of the OR estimates, and the statistical significance of the association was only marginally weaker, suggesting that the observed association is robust to random error. When pooled with the original AJ GWAS data [7], the association was stronger (OR = 1.24, 95% CI 1.13–1.36, p<0.001); however, for the purpose of independent validation in this study, these original “discovery” data were excluded from the current analysis.

Another potential concern related to accurate estimate and adjustment of between-study heterogeneity is the selection of a statistical model of meta-analysis. In the current study, we report the results of fixed-effect analysis as opposed to random effect model. Because of the assumption of low-penetrant effect uniformly correlated with the response, in the context of the current meta-analysis of breast cancer case-control studies, the effect is likely to be similar among the analyzed centers. In addition, for the small underpowered centers likely subjected to random error, overweighing in random model may negatively impact the accuracy of pooled risk assessment. Therefore, a fixed effect model was utilized in the present analysis. A parallel analysis using the random effect models and the results provided were closely similar results (OR = 1.02, 95% CI 0.98–1.06), with the between study heterogeneity of p = 0.005.

We have investigated if other factors also contribute to the heterogeneity of risk estimates observed in this large combined study. As illustrated in the Supporting Information S1, based on the age distribution of cases (median age), we found that the cases from centers with inverse risk estimates were on average 6–17 years younger than the cases from centers showing a susceptible effect. Based on this observation it is possible that the age difference, likely attributable to center ascertainments (e.g. prevalence of familial versus sporadic cases or clinical versus population – based ascertainments), may also influence the 6q22.33 breast cancer risk estimates. For example, one of the outlier studies (kConFab) is predominantly a familial-based ascertainment, with cases and controls on average ∼17 years younger compared to some other studies. This may suggest that ascertainment differences may possibly contribute to the observed heterogeneity, although the recent BCAC studies on other low-penetrant breast cancer GWAS loci suggest such effects to be marginal. In the present study, however, the age stratified analysis revealed the association of 6q22.33 with breast cancer risk (per-allele ORs = 1.05) in the subsets of breast cancer cases >50 years of age, as shown in Table 3 (p for interaction = 0.044) with the strongest effect in the age group of 50–59 under the recessive model (OR = 1.21, 96% CI 1.06–1.38; p-value = 0.006). This suggests that the breast cancer risk attributed to 6q22.33 allele may be slightly modified by age, and hence some source of potential heterogeneity in the risk estimates may stem from the age distribution related to ascertainment differences between “younger” (e.g. kConFab) and “older” (e.g. MSKCC, SEARCH) studies. While the current study does not provide sufficient power to allow for age-specific meta-analysis, this interaction can be thoroughly examined with expansion of larger datasets.

The initial reports of this locus suggested a stronger association for rs2180341 with ER-positive tumors than ER-negative tumors [8]. We did not replicate this finding in the current study (Table 3), although there was weak evidence of an association with risk in ER-positive tumors (per allele OR = 1.04) and no association for risk in ER-negative tumors (per allele OR = 0.99). Other histo-pathological variables may also influence the risk effect of rs2180341, and contribute to the observed heterogeneity. While such scenario is possible, recent studies in BCAC have shown that besides ER/PR status, the interaction of low-penetarnt alleles from GWAS with other tumor characteristics is weaker [19], thus it is unlikely these would substantially impact the observed heterogeneity. With the small effect of rs2180341 and the current size of the present study it was not possible to test the potential interaction of other tumor clinico-pathological factors. Moreover, for many of the sub-studies used in the current meta-analysis, this information was not available, and hence the reduction of power of such partial analysis may impact the pooled association estimates.

Lastly, our study provides the first estimate of the potential breast cancer modifying effect of 6q22.33 in carriers of *BRCA1* mutations. In the 3,361 *BRCA1* mutation carriers in CIMBA, we observed a statistically significant inverse association with breast cancer risk (per-allele HR = 0.89, 95% CI 0.80–1.00, p = 0.048, Table 4, Figure 2A). While this finding may be due to random effects, we note that OR estimates less than one were observed in eight of the ten studies. The two remaining studies from this analysis (PISA and EMBRACE) demonstrated HR greater than one. Fluctuations in the study-specific risk estimate may be due to differences in ascertainment bias (e.g., oversampling of familial cases, selection of younger versus older cases or local population differences) between studies. As the majority of *BRCA1* cancers are ER-, there is also recent evidence suggesting that E3 ubiquitin-ligases (related family of *RNF146*, a candidate gene in 6q22.33) and *BRCA1* may act in conjunction to regulate ER-mediated pathways in breast cancer tumorigenesis [20], [21]. Most interestingly, several recent studies discovered *RNF146* to be a critical player in *Wnt* signaling pathway, providing an evidence for novel biochemical properties of the enzyme in ubiquitination of axin, a critical protein involved in stabilization of beta-catenin [22], [23], [24]. Besides breast tumorigeneis, this significant observation may suggest a broader role of *RNF146* in other types of common cancer.

Hence, further functional analysis is needed to link rs2180341 with tumorigenesis. In our original discovery study, we have demonstrated that rs2180341 tags relatively conserved region of ∼100 kb [7]. In the subsequent study [8], our preliminary data indicated a trend of increased expression of *RNF146* with the dosage of high-risk allele of rs2180341. While sequencing of the subset of breast tumors did not identify any coding SNPs in *RNF146* associated with the risk allele [8], it is likely that there are other non-coding variants correlated with rs2180341 that may explain observed genotype/expression trend. Using the data from recent release of 1000 genomes we have identified several SNPs highly correlated with rs2180341; 2 of them with significantly predictive functional impact on putative transcription binding sites (Supporting Information S1). Interestingly, rs2180341 maps in a histone mark region, identified by CHIP-seq (Supporting Information S1), suggesting potential involvement of these variants in regulation of the expression of nearby genes, including *RNF146.* In order to provide further biological insight, the more systematic analysis would be needed to test the correlation of *RNF146* expression with identified genetic variants in larger subset of breast tumors.

In conclusion, this large study found evidence for a weak overall association between the 6q22.33 locus and sporadic breast cancer risk. Relative risk estimates for rs2180341 were lower in non-AJ Europeans as compared to AJ populations. The study illustrates the difficulties inherent in the reliable estimation of low risk susceptibility alleles – even with a study as large as the current one, in which the overall effect was only marginally significant. It is likely there are many such variants, conferring ORs<1.1, and characterizing such associations with common diseases disease presents a major challenge. It is possible that comprehensive sequencing across the region may identify the true causal variant(s) with stronger effects. If the heterogeneity among studies is due to differences in linkage disequilibrium (LD), fine-scale mapping might also allow identification of more strongly associated variants. The combined effect of these common variants and other as-yet-undiscovered rare variants, together with lifestyle risk factors, could provide the basis for risk algorithms for the preventive management of breast cancer.

## Supporting Information

Funding S1 **The description of sources of funding for the study in detail.**
(DOCX)

Supporting Information S1 **Table A: Summary of the 25 breast cancer case studies used in the BCAC analyses Table B: Genotype frequency among Caucasian BCAC case and controls, minor allele frequencies (MAF), and Hardy-Weinberg Equilibrium (HWE) by study Table C: Summary of the 11 breast cancer case studies used in the CIMBA analyses Table D: Genotype frequency among CIMBA case and controls, minor allele frequencies (MAF), and Hardy-Weinberg Equilibrium (HWE) by study Table E: Ethics committee approvals (IRB approvals) Table F: SNPs from 6q22.33 with the highest functional impact and highly correlated with rs2180341. Correlated proxies of rs2180341 (r2>0.8) were extracted from latest release of 1000 genomes project on ~300 individuals of European ancestry. The functional impact of all correlated SNPs was assessed using the pipelines of ANNOVAR suite. The functional impact (FI) of each SNP for each of 3 selected categories is defined by FI score. TF binding site prediction also includes DNAI hypersensitivity data. Conserved elements were assessed using placental 46way analysis. r-square values are relative to rs2180341.**
(DOC)
