# Supplementary material for: Breast Cancer Risk and 6q22.33: Combined Results from Breast Cancer Association Consortium and Consortium of Investigators on Modifiers of BRCA1/2
Source: PLoS One. 2012 Jun 29;7(6):e35706. doi: 10.1371/journal.pone.0035706 (PMC3387216; doi:10.1371/journal.pone.0035706)
Supplement: Funding S1 — The description of sources of funding for the study in detail. (DOCX) [file pone.0035706.s001.docx]

**FUNDING INFORMATION**

*This study was supported by The Breast Cancer Research Foundation (K.O., T.K.) and:*

*BCAC:*

The MCBCS was supported by the National Institutes of Health grant CA122340 and a Sponsored Program of Research Excellence (SPORE) grant in Breast Cancer P50 CA116201.

The *KBCP* has been financially supported by the Kuopio University Hospital EVO Research Fund, Academy of Finland, the Finnish Cancer Society, the University of Kuopio and EVO research funding of Vaasa Hospital District.

The KARBAC was supported through the regional agreement on medical training and clinical research (ALF) between Stockholm County Council and Karolinska Institute, from The Swedish Cancer Society, Bert von Kantzow Foundation, and Gustav V Jubilee Foundation.

The HABCS was supported by an intramural grant of Hannover Medical School. NB was generously supported by the Friends of Hannover Medical School.

The *LMBC* is supported by European Union Framework Programme 6 Project LSHC-CT-2003-503297 (the Cancerdegradome) and by the ‘Stichting tegen Kanker’ (232-2008).

The *MSKCC* was supported by Breast Cancer Research Foundation, Niehaus Clinical Cancer Genetics Initiative, Andrew Sabin Family Foundation and Lymphoma Foundation and National Institute of Health (5R21 CA139396-02, TK)

The *MCCS* receives its core funding from the Cancer Council Victoria and is supported by grants from the NHMRC (209057, 251533, 396414, 504711, and 504715).

The GC-HBOC study was supported by Deutsche Krebshilfe (107054), by the Center of Molecular Medicine, Cologne, and the Helmholtz society.

*GENICA* Network was funded by the Federal Ministry of Education and Research (BMBF) Germany grants 01KW9975/5, 01KW9976/8, 01KW9977/0 and 01KW0114, the Robert Bosch Foundation of Medical Research, Stuttgart, Deutsches Krebsforschungszentrum (DKFZ) Heidelberg, Institute for Prevention and Occupational Medicine of the German Social Accident Insurance (IPA), Bochum, as well as the Department of Internal Medicine, Evangelische Kliniken Bonn gGmbH, Johanniter Krankenhaus, Bonn, Germany.

The CNIO-BCS was partly funded by the Red Temática de Investigación Cooperativa en Cáncer, the Asociación Española Contra Cáncer and grants from the Fondo de Investigación Santiario (PI081583 to R.L.M. and PI081120 to J.B.).

The *CGPS* was supported by the Neye Foundation. Chief Physician Johan Boserup and Lise Boserup Fund, the Danish Medical Research Council and Copenhagen University Hospital, Herlev Hospital.

The *BBCS* and Mammography Oestrogens and Growth Factors studies are funded by Cancer Research UK and Breakthrough Breast Cancer, with NHS funding to the NIHR Biomedical Research Centre and the National Cancer Research Network (NCRN).

The *ABCS* study was supported by the Dutch Cancer Society [grants NKI 2001-2423; 2007-3839] and the Dutch National Genomics Initiative.

The ABCFS was funded by the National Health and Medical Research Council, the Victorian Health Promotion Foundation, the New South Wales Cancer Council, and as part of the Breast Cancer Family Registry funded by the National Cancer Institute (USA) under RFA # CA-95-003.

The SBCS was supported by Yorkshire Cancer Research and the Breast Cancer Campaign.

SEARCH was funded by Cancer Research-UK (CR-UK); contributing authors, PDPP is a Senior Clinical Research Fellow.

The SEBCS was supported by a grant from the National Research and Development Program for Cancer Control, Ministry of Health and Welfare, Republic of Korea (0620410-1).

The TWBCS was supported by the Department of Health, Taiwan. UCIBCS is supported by grants from the NCI (CA2 R01 CA58860-14) and the Lon v smith Foundation (LVSF-41027, 3834, 42344).

The kConFab is supported by grants from the National Breast Cancer Foundation, the National Health and Medical Research Council (NHMRC) and by the Queensland Cancer Fund, the Cancer Councils of New South Wales, Victoria, Tasmania and South Australia, and the Cancer Foundation of Western Australia. Financial support was provided by: U.S. Army Medical Research and Materiel Command under DAMD17-01-1-0729, the Cancer Council Tasmania and Cancer Foundation of Western Australia (AOCS study); The National Health and Medical Research Council of Australia (199600) (ACS study). The content of this manuscript does not necessarily reflect the views or policies of the National Cancer Institute or any of the collaborating centers in the CFRs, nor does mention of trade names, commercial products, or organizations imply endorsement by the U.S. Government or the CFRs Centers. The genotyping and analysis were supported by grants from the National Health and Medical Research Council (NHMRC). ABS was funded by an NHMRC Career Development Award, and GC-T and JLH are NHMRC Senior Principal Research Fellows.

*CIMBA:*

*The CIMBA data management and coordination is supported by Cancer Research UK. ACA is a Cancer Research UK, Senior Cancer Research Fellow.*

*The Kathleen Cunningham Consortium for Research into Familial Breast Cancer (kConFab):* kConFab is supported by grants from the National Breast Cancer Foundation, the National Health and Medical Research Council (NHMRC) and by the Queensland Cancer Fund, the Cancer Councils of New South Wales, Victoria, Tasmania and South Australia, and the Cancer Foundation of Western Australia. ABS is an NHMRC Senior Research Fellow, and GCT is an NHMRC Senior Principal Research Fellow.

*MAYO study* is supported in part by National Institute of Health Grants CA116167, CA128978, a grant from the Breast Cancer Research Foundation, and a grant from the Komen Foundation for the Cure.

The *HEBCS* study has been financially supported by the Helsinki University Central Hospital Research Fund, Academy of Finland (132473), the Finnish Cancer Society and the Sigrid Juselius Foundation.

*UPENN study* is supported by the Breast Cancer Research Foundation.

*The Hereditary Breast and Ovarian Cancer Research Group Netherlands (HEBON)*: The HEBON study is supported by the Dutch Cancer Society grants NKI 1998-1854, NKI 2004-3088 and NKI 2007-3756.

*Epidemiological study of BRCA1* and *BRCA2 mutation carriers (EMBRACE):* EMBRACE is funded by Cancer Research-UK Grants C1287/A10118 and C1287/A8874. Craig Luccarini is funded by Cancer Research-UK Grants C8197/A10123 and C8197/A10865. D.Gareth Evans and Fiona Lalloo are supported by an NIHR grant to the Biomedical Research Centre, Manchester. The Investigators at The Institute of Cancer Research and The Royal Marsden NHS Foundation Trust are supported by an NIHR grant to the Biomedical Research Centre at The Institute of Cancer Research and The Royal Marsden NHS Foundation Trust. Ros Eeles, Elizabeth Bancroft and Lucia D’Mello are also supported by Cancer Research UK Grant C5047/A8385.

The *ILUH* is supported by the Research Fund of Landspitali-University Hospital and the Icelandic association: “Walking for Breast Cancer Research”.

*GEORGETOWN*: Claudine Isaacs, Fisher Center for Familial Cancer Research, Lombardi Cancer Center, Georgetown University, Washington, DC, USA (CI).

AC Antoniou is a Cancer Research UK Senior Cancer Research Fellow, L McGuffog, the CIMBA genotyping and data management are funded by Cancer Research UK.
