# Supplementary material for: Breast Cancer Risk and 6q22.33: Combined Results from Breast Cancer Association Consortium and Consortium of Investigators on Modifiers of BRCA1/2
Source: PLoS One. 2012 Jun 29;7(6):e35706. doi: 10.1371/journal.pone.0035706 (PMC3387216; doi:10.1371/journal.pone.0035706)
Supplement: Supporting Information S1 — Table A: Summary of the 25 breast cancer case studies used in the BCAC analyses Table B: Genotype frequency among Caucasian BCAC case and controls, minor allele frequencies (MAF), and Hardy-Weinberg Equilibrium (HWE) by study Table C: Summary of the 11 breast cancer case studies used in the CIMBA analyses Table D: Genotype frequency among CIMBA case and controls, minor allele frequencies (MAF), and Hardy-Weinberg Equilibrium (HWE) by study Table E: Ethics committee approvals (IRB approvals) Table F: SNPs from 6q22.33 with the highest functional impact and highly correlated with rs2180341. Correlated proxies of rs2180341 (r2>0.8) were extracted from latest release of 1000 genomes project on ~300 individuals of European ancestry. The functional impact of all correlated SNPs was assessed using the pipelines of ANNOVAR suite. The functional impact (FI) of each SNP for each of 3 selected categories is defined by FI score. TF binding site prediction also includes DNAI hypersensitivity data. Conserved elements were assessed using placental 46way analysis. r-square values are relative to rs2180341. (DOC) [file pone.0035706.s002.doc]

**SUPPORTING INFORMATION**

**Table A. Summary of the 25 breast cancer case studies used in the BCAC analyses**

| Study | Abbreviation | References^1^ | Location of Study; Description of Study Design | Controls  (median age) | Invasive cases  (median age) | Age range (cases) | | | Ethnicity  (E: European origin; A: Asian origin) | Genotyping platform(s)^2^ |
| --- | --- | --- | --- | --- | --- | --- | --- | --- | --- | --- |
| Australian Breast Cancer Family Study | ABCFS | (1) | Melbourne and Sydney, Australia; population-based case-control-family study | 628(40) | 1,138 (43) | 20 | - | 68 | E | Taqman/ Sequenom iPLEX |
| Amsterdam Breast Cancer Study | ABCS | (2-4) | Amsterdam, The Netherlands; retrospective cohort of cases, population-based controls | 548 (36) | 1,404 (44) | 23 | - | 50 | E | Taqman |
| 1.) British Breast Cancer Study (cases and controls)  2.) Mammography Oestrogens and Growth Factors Study (controls) | BBCS | (5, 6) | 1.) English and Scottish Registries  2.) London, England; randomized controlled trial of mammographic screening | 804 (55) | 1,106 (56) | 25 | - | 70 | E | Taqman |
| Copenhagen Breast Cancer Study and Copenhagen General Population Study | CGPS | (7, 8) | Denmark (Copenhagen); population-based | 6,652 (55) | 2,005 (62) | 20 | - | 93 | E | Taqman |
| Spanish National Cancer Centre Breast Cancer Study | CNIO-BCS | (9) | Spain; hospital-based case-control study | 815 (53) | 704 (54) | 23 | - | 86 | E | Taqman |
| ICR Familial Breast Cancer Study | FBCS | (10) | United Kingdom; cases: clinic-based recruitment of familial breast cancer patients. controls: population-based | 1,023 (NA) | 882 (47) | 18 | - | 87 | E | Taqman |
| German Consortium for Hereditary Breast and Ovarian Cancer | GC-HBOC | (11) | Germany; familial case-control study | 1,218 (52) | 850 (46) | 19 | - | 87 | E | Taqman |
| Gene Environment Interaction and Breast Cancer in Germany | GENICA | (12, 13) | Germany; population-based case-control study | 1,012 (59) | 1,016 (59) | 23 | - | 80 | E | Taqman |
| Genetic Epidemiology Study of Breast Cancer by Age 50 | GESBC | (14) | Germany; population-based case-control study | 551 (44) | 511 (43) | 24 | - | 50 | E | Taqman |
| Hannover Breast Cancer Study | HABCS | (15) | Germany; hospital-based | 1,014 (29) | 1,044 (57) | 27 | - | 91 | E (99.9%) | Taqman |
| Hannover-Minsk Breast Cancer Study | HMBCS | (16) | Belarus; hospital-based cases and population-based controls | 1,017 (38) | 1,762 (48) | 16 | - | 82 | E | Taqman |
| Hannover-Ufa Breast Cancer Study | HUBCS | (16) | Russia; hospital-based cases and population-based controls | 982 (NA) | 730 (51) | 26 | - | 85 | E | Taqman |
| Karolinska Breast Cancer Study | KARBAC | (17, 18) | Stockholm, Sweden; blood-donors controls, familial and population-based cases | 844 (NA) | 805 (56) | 24 | - | 88 | E | Taqman |
| Kuopio Breast Cancer Project | KBCP | (19) | Finland; hospital-based prospective clinical cohort | 389 (53) | 464 (56) | 17 | - | 92 | E | Taqman |
| Katholieke Universiteit Leuven | LMBC | (20, 21) | Belgium; hospital-based case-control study | 1,090 (45) | 645 (54) | 19 | - | 89 | E | Taqman |
| Mayo Clinic Breast Cancer Study | MCBCS | (22) | US; clinic-based case-control study | 1,252 (59) | 1,147 (56) | 20 | - | 90 | E (99.5%) | Taqman |
| Melbourne Collaborative Cohort Study | MCCS | (23) | Australia: population-based prospective cohort study | 757 (58) | 665 (62) | 41 | - | 83 | E | Taqman |
| Memorial Sloan-Kettering Cancer Center Study | MSKCC | (24) | US; hospital-based case-control study | 1,600 (54) | 1,782 (54) | 20 | - | 91 | E | Taqman |
| Ontario Familial Breast Cancer Registry | OFBCR | (25) | Population-based familial case-control study | 365 (56) | 1,358 (54) | 22 | - | 81 | E | Taqman |
| Leiden University Medical Centre Breast Cancer Study | ORIGO | (26) | Netherlands (Leiden and Rotterdam), hospital-based | 1,135 (52) | 576 (52) | 21 | - | 87 | E | Taqman |
| Sheffield Breast Cancer Study | SBCS | (27, 28) | England; hospital-based case-control study | 1,193 (58) | 1,059 (59) | 28 | - | 92 | E | Taqman |
| Study of Epidemiology and Risk factors in Cancer Heredity | SEARCH | (29) | England; population-based case-control study | 6,719 (60) | 6,507 (53) | 23 | - | 81 | E | Taqman |
| Seoul Breast Cancer Study | SEBCS | (30, 31) | Seoul, Korea; hospital-based case-controls study | 1,115 (50) | 1,688 (48) | 22 | - | 82 | A | Taqman |
| Taiwanese Breast Cancer Study | TWBCS | (32) | Taiwan; hospital-based case-control study | 928 (46) | 894(50) | 18 | - | 99 | A | Sequenom iPLEX |
| Kathleen Cuningham Foundation Consortium for Familial Breast Cancer/ Australian Ovarian Cancer Study | KConFab/ AOCS | (33) | Australia and New Zealand; clinic-based recruitment of familial breast cancer patients (cases) | 663 (58) | 459 (44) | 20 | - | 81 | E | Sequenom iPLEX |
|  |  |  | Australia; population-based case-control study of ovarian cancer and population-based cancer-family study of breast cancer (controls) |  |  |  |  |  |  |  |

^1^References (below)

^2^ Most genotyping was conducted using Taqman assays (Applied Biosystems, Foster City, CA), although some studies used the iPLEX Sequenom MassArray system (Sequenom Inc., San Diego, CA) or PCR-based RFLP (study-specific platforms are detailed in Table A). Genotyping was conducted at each institution with the exception of samples from KARBAC, ORIGO, SEBCS, and UCIBCS, which were genotyped at Strangeways Research Laboratories (Cambridge, England). In addition to study participants, most laboratories genotyped a common set of 90 CEPH trio DNAs used by HapMap (HAPMAPPT01, Coriell Institute for Medical Research, Cambden, NJ) for QC purposes.

| **Table B.** Genotype frequency among Caucasian BCAC case and controls, minor allele frequencies (MAF), and Hardy-Weinberg Equilibrium (HWE) by study | | | | | | | | | | |
| --- | --- | --- | --- | --- | --- | --- | --- | --- | --- | --- |
|  | **Cases** | | | | **Controls** | | | |  | **HWE  p-value** |
| **Study** | **AA** | **AG** | **GG** | **Total** | **AA** | **AG** | **GG** | **Total** | **MAF** |
| ABCFS | 635 | 431 | 72 | 1,138 | 363 | 228 | 37 | 628 | 0.24 | 0.88 |
| ABCS | 781 | 521 | 102 | 1,404 | 300 | 213 | 35 | 548 | 0.26 | 0.73 |
| BBCS | 605 | 414 | 87 | 1,106 | 441 | 317 | 46 | 804 | 0.25 | 0.26 |
| CGPS | 1,146 | 748 | 111 | 2,005 | 3,788 | 2,477 | 387 | 6,652 | 0.24 | 0.50 |
| CNIO-BCS | 412 | 246 | 46 | 704 | 453 | 319 | 43 | 815 | 0.25 | 0.17 |
| FBCS | 482 | 340 | 60 | 882 | 564 | 386 | 73 | 1,023 | 0.26 | 0.53 |
| GC-HBOC | 478 | 308 | 64 | 850 | 684 | 453 | 81 | 1,218 | 0.25 | 0.61 |
| GENICA | 556 | 385 | 75 | 1,016 | 573 | 382 | 57 | 1,012 | 0.25 | 0.52 |
| GESBC | 281 | 207 | 23 | 511 | 289 | 229 | 33 | 551 | 0.27 | 0.16 |
| HABCS | 564 | 399 | 81 | 1,044 | 559 | 390 | 65 | 1,014 | 0.26 | 0.78 |
| HMBCS | 1,023 | 625 | 114 | 1,762 | 527 | 421 | 69 | 1,017 | 0.27 | 0.22 |
| HUBCS | 374 | 301 | 55 | 730 | 542 | 369 | 71 | 982 | 0.26 | 0.45 |
| KARBAC | 471 | 299 | 35 | 805 | 482 | 311 | 51 | 844 | 0.24 | 0.93 |
| KBCP | 236 | 197 | 31 | 464 | 205 | 145 | 39 | 389 | 0.29 | 0.08 |
| LMBC | 297 | 308 | 40 | 645 | 547 | 461 | 82 | 1,090 | 0.29 | 0.26 |
| MCBCS | 652 | 422 | 73 | 1,147 | 707 | 473 | 72 | 1,252 | 0.25 | 0.54 |
| MCCS | 367 | 260 | 38 | 665 | 432 | 292 | 33 | 757 | 0.24 | 0.060 |
| MSKCC | 1,562 | 1,135 | 215 | 2,912 | 1,855 | 1,082 | 158 | 3,095 | 0.23 | 0.99 |
| OFBCR | 786 | 489 | 83 | 1,358 | 203 | 147 | 15 | 365 | 0.24 | 0.066 |
| ORIGO | 330 | 208 | 38 | 576 | 638 | 419 | 78 | 1,135 | 0.25 | 0.42 |
| SBCS | 581 | 405 | 73 | 1,059 | 707 | 428 | 58 | 1,193 | 0.23 | 0.51 |
| SEARCH | 3,566 | 2,556 | 385 | 6,507 | 3,848 | 2,488 | 383 | 6,719 | 0.24 | 0.47 |
| SEBCS | 888 | 658 | 142 | 1,688 | 644 | 425 | 86 | 1,155 | 0.26 | 0.17 |
| TWBCS | 578 | 285 | 31 | 894 | 548 | 336 | 44 | 928 | 0.23 | 0.41 |
| Kconfab/AOCS | 286 | 154 | 19 | 459 | 365 | 258 | 40 | 663 | 0.25 | 0.53 |

**Table C. Summary of the 11 breast cancer case studies used in the CIMBA analyses**

| Study | Abbreviation | References^1^ | Country of Study; Description of Study Design | Unaffected BRCA1/2 Carriers | Affected BRCA1/2 Carriers | Age (cases) range | | | Ethnicity  (E: European origin; A: Asian origin) |
| --- | --- | --- | --- | --- | --- | --- | --- | --- | --- |
| The Netherlands Collaborative Group on Hereditary Breast Cancer | DNA HEBON | (34) | Netherlands; | 622 | 523 | 19 | - | 72 | E |
| Epidemiological Study of BRCA1 and BRCA2 mutation carriers | EMBRACE | (35) | UK & Eire; hospital-based ascertainment of participants from clinical genetics centers | 748 | 772 | 20 | - | 71 | E |
| Fox Chase Cancer Center | FCCC | (36) | USA; | 85 | 59 | 28 | - | 78 | E |
| Georgetown University | GEORGETOWN |  | USA; | 25 | 30 | 28 | - | 62 | E |
| Helsinki Breast Cancer Study | HEBCS |  | Finland; | 91 | 116 | 25 | - | 77 | E |
| Iceland Landspitali - University Hospital | ILUH |  | Iceland; | 13 | 80 | 30 | - | 76 | E |
| Kathleen Cuningham Foundation Consortium for Familial Breast Cancer | kConFab | (33) | Australia and New Zealand; clinic-based recruitment of familial breast cancer patients (cases) | 437 | 546 | 23 | - | 85 | E |
| Mayo Clinic | MAYO |  | USA; | 154 | 186 | 22 | - | 71 | E |
| Università di Pisa | PISA |  | Italy; | 45 | 75 | 23 | - | 74 | E |
| Swedish Breast Cancer Study | SWE-BRCA |  | Sweden; | 347 | 284 | 21 | - | 81 | E |
| University of Pennsylvania | UPENN |  | USA; | 156 | 265 | 23 | - | 85 | E |

| **Table D.** Genotype frequency among CIMBA case and controls, minor allele frequencies (MAF), and Hardy-Weinberg Equilibrium (HWE) by study | | | | | | | | | | |
| --- | --- | --- | --- | --- | --- | --- | --- | --- | --- | --- |
| Study | BRCA1/2 affected carriers | | | | BRCA1/2 unaffected carriers | | | | | |
|  | AA | AG | GG | Total | AA | AG | GG | Total | MAF | HWE^1^ |
| DNA HEBON | 298 | 160 | 29 | 487 | 334 | 222 | 32 | 588 | 24.3 | 0.53 |
| EMBRACE | 445 | 262 | 41 | 748 | 431 | 257 | 42 | 730 | 23.4 | 0.65 |
| FCCC | 32 | 17 | 3 | 52 | 42 | 30 | 6 | 78 | 26.9 | 0.84 |
| GEORGETOWN | 13 | 11 | 2 | 26 | 12 | 8 | 4 | 24 | 33.3 | 0.22 |
| HEBCS | 68 | 38 | 10 | 116 | 44 | 39 | 8 | 91 | 30.2 | 0.88 |
| ILUH | 38 | 38 | 3 | 79 | 8 | 5 | 0 | 13 | 19.2 | 0.39 |
| Kconfab | 306 | 173 | 30 | 509 | 225 | 159 | 29 | 413 | 26.3 | 0.90 |
| MAYO | 108 | 64 | 6 | 178 | 93 | 49 | 9 | 151 | 22.2 | 0.46 |
| PISA | 45 | 25 | 3 | 73 | 24 | 20 | 0 | 44 | 22.7 | 0.051 |
| SWE-BRCA | 162 | 89 | 17 | 268 | 175 | 137 | 19 | 331 | 26.4 | 0.24 |
| UPENN | 131 | 89 | 20 | 240 | 74 | 60 | 8 | 142 | 26.8 | 0.35 |
| ^1^Genotype frequencies for unaffected carriers were evaluated for HWE proportions using χ2 test (1 d.f) | | | | | | | | | | |

| **Table E.** Ethics committee approvals (IRB approvals) | | |
| --- | --- | --- |
| **Abbreviations** | **Country** | **Committee approval** |
| **BCAC** |  |  |
| ABCFS | Australia | The University of Melbourne Health Sciences Human Ethics Sub-Committee (HESC) |
| ABCS | Netherlands | LUMC Commissie Medische Ethiek and Protocol Toetsingscommissie van het NKI/Antoni van Leeuwenhoek Ziekenhuis |
| BBCS | UK | South East Multi-Centre Research Ethics Committee |
| CGPS | Denmark | Kobenhavns Amt den Videnskabsetiske Komite |
| CNIO-BCS | Spain | Hospital Universitario La Paz Comite Etico de Investigacion Clinica |
| FBCS | UK | The London Multi-Centre Research Ethics Committees |
| GC-HBOC | Germany | Ethik-Kommission der Medizinischen Fakultat der Universitat zu Koln |
| GENICA | Germany | Rheinische Friedrich-Wilhelms-Universitat Medizinische Einrichtungen Ethik-Kommission |
| GESBC | Germany | Ruprecht-Karls-Universitat Medizinische Fakultat Heidelberg Ethikkommission |
| HABCS | Germany | Medizinische Hochschule Hannover Ethik-Kommission |
| HMBCS | Belarus | Medizinische Hochschule Hannover Ethik-Kommission |
| HUBCS | Russia | Ethical Committee of Institute of Biochemistry and Genetics Ufa Science Center |
| KARBAC | Sweden | Lokala Forskningsetikkommitten Nord |
| KBCP | Finland | Pohjois-Savon Sairraanhoitopiirin Kuntayhtyma Tutkimuseettinen Toimikunta |
| kConFab/AOCS | Australia | The Queenland Institute of Medical Research Human Research Ethics Committee (QIMR-HREC) |
| LMBC | Belgium | Commissie Medische Ethiek van de Universitaire Ziekenhuizen Kuleuven |
| MCBCS | USA | Mayo Clinic IRB |
| MCCS | Australia | The Cancer Council Victoria Human Research Ethics Committee |
| MSKCC | USA | Memorial Sloan-Kettering Cancer Center IRB |
| OFBCR | Canada | Mount Sinai Hospital Research Ethics Board |
| ORIGO | Netherlands | Medical Ethical Committee and Board of Directors of the Leiden University Medical Center (LUMC) |
| SBCS | UK | South Sheffield Research Ethics Committee |
| SEARCH | UK | Multi Centre Research Ethics Committee (MREC) |
| SEBCS | Korea | Seoul National University College of Medicine/Seoul National University Hospital IRB |
| TWBCS | Taiwan | Human Subject Research Ethics Committee/IRB Academia Sinica |
|  |  |  |
| **CIMBA** |  |  |
| DNA-HEBON | The Netherlands | Protocol Toetsingscommissie van het NKI/Antoni van Leeuwenhoek Ziekenhuis |
| EMBRACE | UK and EIRE | Anglia & Oxford MREC |
| FCCC | USA | Institutional Review Board Fox Chase Cancer Center |
| GEORGETOWN | USA | MedStar Research Institute - Georgetown University Oncology Institutional Review Board |
| HEBCS | Finland | Helsingin ja uudenmaan sairaanhoitopiiri (Helsinki University Central Hospital ethics committee) |
| ILUH | Iceland | Vísindasiđanefnd National Boethics Committee |
| KCONFAB | Australia | Peter MacCallum Cancer Centre Ethics Committee |
| KCONFAB | Australia | Queensland Institute of Medical Research - Human Research Ethics Committee |
| MAYO | USA | Mayo Clinic Institutional Review Boards |
| MSKCC | USA | Memorial Sloan-Kettering Cancer Center IRB |
| MSKCC | USA | Human Biospecimen Utilization Committee |
| SWE-BRCA | Sweden | Regionala Etikprövningsnämnden Stockholm |
| UPENN | USA | University of Pennsylvania Institutional Review Board |
|  |  |  |

**Table F.** SNPs from 6q22.33 with the highest functional impact and highly correlated with rs2180341

Correlated proxies of rs2180341 (r2>0.8) were extracted from latest release of 1000 genomes project on ~300 individuals of European ancestry. The functional impact of all correlated SNPs was assessed using the pipelines of ANNOVAR suite. The functional impact (FI) of each SNP for each of 3 selected categories is defined by FI score. TF binding site prediction also include DNAI hypersensitivity data. Conserved elements were assessed using placental 46way analysis. r-square values are relative to rs2180341.

**SUPPORTING INFORMATION REFERENCES**

1. Spurdle, A. B., Hopper, J. L., Chen, X., et al. The progesterone receptor exon 4 Val660Leu G/T polymorphism and risk of breast cancer in Australian women. Cancer Epidemiol Biomarkers Prev 2002; 11*:* 439-43.

2. Schmidt, M. K., Tollenaar, R. A., de Kemp, S. R., et al. Breast cancer survival and tumor characteristics in premenopausal women carrying the CHEK2*1100delC germline mutation. J Clin Oncol 2007; 25*:* 64-9.

3. Verschuren, W., Van Leer, E., Blokstra, A., et al. Cardiovascular disease risk factors in The Netherlands. . Neth J Cardiol 1993; 4*:* 205-210.

4. Blokstra, A., Smit, H., Bueno-de-Mesquita, H., Seidell, J., and Verschuren, W. Monitoring van Risicofactoren en Gezondheid in Nederland (MORGEN-project), 1993-1997. Leefstijl- en risicofactoren: prevalenties en trends. Bilthoven: RIVM, 2005.

5. Johnson, N., Fletcher, O., Naceur-Lombardelli, C., et al. Interaction between CHEK2*1100delC and other low-penetrance breast-cancer susceptibility genes: a familial study. Lancet 2005; 366*:* 1554-7.

6. Fletcher, O., Johnson, N., Palles, C., et al. Inconsistent association between the STK15 F31I genetic polymorphism and breast cancer risk. J Natl Cancer Inst 2006; 98*:* 1014-8.

7. Bojesen, S. E., Tybjaerg-Hansen, A., Axelsson, C. K., and Nordestgaard, B. G. No association of breast cancer risk with integrin beta3 (ITGB3) Leu33Pro genotype. Br J Cancer 2005; 93*:* 167-71.

8. Weischer, M., Bojesen, S. E., Tybjaerg-Hansen, A., Axelsson, C. K., and Nordestgaard, B. G. Increased risk of breast cancer associated with CHEK2*1100delC. J Clin Oncol 2007; 25*:* 57-63.

9. Milne, R. L., Ribas, G., Gonzalez-Neira, A., et al. ERCC4 associated with breast cancer risk: a two-stage case-control study using high-throughput genotyping. Cancer Res 2006; 66*:* 9420-7.

10. Seal, S., Thompson, D., Renwick, A., et al. Truncating mutations in the Fanconi anemia J gene BRIP1 are low-penetrance breast cancer susceptibility alleles. Nat Genet 2006; 38*:* 1239-41.

11. Frank, B., Hemminki, K., Wappenschmidt, B., et al. Association of the CASP10 V410I variant with reduced familial breast cancer risk and interaction with the CASP8 D302H variant. Carcinogenesis 2006; 27*:* 606-9.

12. Pesch, B., Ko, Y., Brauch, H., et al. Factors modifying the association between hormone-replacement therapy and breast cancer risk. Eur J Epidemiol 2005; 20*:* 699-711.

13. Justenhoven, C., Pierl, C. B., Haas, S., et al. The CYP1B1_1358_GG genotype is associated with estrogen receptor-negative breast cancer. Breast Cancer Res Treat 2008; 111*:* 171-7.

14. Chang-Claude, J., Eby, N., Kiechle, M., Bastert, G., and Becher, H. Breastfeeding and breast cancer risk by age 50 among women in Germany. Cancer Causes Control 2000; 11*:* 687-95.

15. Dork, T., Bendix, R., Bremer, M., et al. Spectrum of ATM gene mutations in a hospital-based series of unselected breast cancer patients. Cancer Res 2001; 61*:* 7608-15.

16. Bogdanova, N., Cybulski, C., Bermisheva, M., et al. A nonsense mutation (E1978X) in the ATM gene is associated with breast cancer. Breast Cancer Res Treat 2009; 118*:* 207-11.

17. Lindblom, A., Rotstein, S., Larsson, C., Nordenskjold, M., and Iselius, L. Hereditary breast cancer in Sweden: a predominance of maternally inherited cases. Breast Cancer Res Treat 1992; 24*:* 159-65.

18. Margolin, S., Werelius, B., Fornander, T., and Lindblom, A. BRCA1 mutations in a population-based study of breast cancer in Stockholm County. Genet Test 2004; 8*:* 127-32.

19. Hartikainen, J. M., Tuhkanen, H., Kataja, V., et al. An autosome-wide scan for linkage disequilibrium-based association in sporadic breast cancer cases in eastern Finland: three candidate regions found. Cancer Epidemiol Biomarkers Prev 2005; 14*:* 75-80.

20. De Maeyer, L., Van Limbergen, E., De Nys, K., et al. Does estrogen receptor negative/progesterone receptor positive breast carcinoma exist? J Clin Oncol 2008; 26*:* 335-6; author reply 336-8.

21. Neven, P., Brouckaert, O., Van Belle, V., et al. In early-stage breast cancer, the estrogen receptor interacts with correlation between human epidermal growth factor receptor 2 status and age at diagnosis, tumor grade, and lymph node involvement. J Clin Oncol 2008; 26*:* 1768-9; author reply 1769-71.

22. Olson, J. E., Ma, C. X., Pelleymounter, L. L., et al. A comprehensive examination of CYP19 variation and breast density. Cancer Epidemiol Biomarkers Prev 2007; 16*:* 623-5.

23. Giles, G. G., and English, D. R. The Melbourne Collaborative Cohort Study. IARC Sci Publ 2002; 156*:* 69-70.

24. Kirchhoff, T., Chen, Z. Q., Gold, B., et al. The 6q22.33 locus and breast cancer susceptibility. Cancer Epidemiol Biomarkers Prev 2009; 18*:* 2468-75.

25. John, E. M., Hopper, J. L., Beck, J. C., et al. The Breast Cancer Family Registry: an infrastructure for cooperative multinational, interdisciplinary and translational studies of the genetic epidemiology of breast cancer. Breast Cancer Res 2004; 6*:* R375-89.

26. de Bock, G. H., Schutte, M., Krol-Warmerdam, E. M., et al. Tumour characteristics and prognosis of breast cancer patients carrying the germline CHEK2*1100delC variant. J Med Genet 2004; 41*:* 731-5.

27. MacPherson, G., Healey, C. S., Teare, M. D., et al. Association of a common variant of the CASP8 gene with reduced risk of breast cancer. J Natl Cancer Inst 2004; 96*:* 1866-9.

28. Rafii, S., O'Regan, P., Xinarianos, G., et al. A potential role for the XRCC2 R188H polymorphic site in DNA-damage repair and breast cancer. Hum Mol Genet 2002; 11*:* 1433-8.

29. Lesueur, F., Pharoah, P. D., Laing, S., et al. Allelic association of the human homologue of the mouse modifier Ptprj with breast cancer. Hum Mol Genet 2005; 14*:* 2349-56.

30. Han, S., Lee, K. M., Choi, J. Y., et al. CASP8 polymorphisms, estrogen and progesterone receptor status, and breast cancer risk. Breast Cancer Res Treat 2007.

31. Lee, K. M., Choi, J. Y., Kang, C., et al. Genetic polymorphisms of selected DNA repair genes, estrogen and progesterone receptor status, and breast cancer risk. Clin Cancer Res 2005; 11*:* 4620-6.

32. Hsu, H. M., Wang, H. C., Chen, S. T., et al. Breast cancer risk is associated with the genes encoding the DNA double-strand break repair Mre11/Rad50/Nbs1 complex. Cancer Epidemiol Biomarkers Prev 2007; 16*:* 2024-32.

33. Mann, G. J., Thorne, H., Balleine, R. L., et al. Analysis of cancer risk and BRCA1 and BRCA2 mutation prevalence in the kConFab familial breast cancer resource. Breast Cancer Res 2006; 8*:* R12.

34. Andrieu, N., Easton, D. F., Chang-Claude, J., et al. Effect of chest X-rays on the risk of breast cancer among BRCA1/2 mutation carriers in the international BRCA1/2 carrier cohort study: a report from the EMBRACE, GENEPSO, GEO-HEBON, and IBCCS Collaborators' Group. J Clin Oncol 2006; 24*:* 3361-6.

35. Spurdle, A. B., Antoniou, A. C., Duffy, D. L., et al. The androgen receptor CAG repeat polymorphism and modification of breast cancer risk in BRCA1 and BRCA2 mutation carriers. Breast Cancer Res 2005; 7*:* R176-83.

36. Rebbeck, T. R., Wang, Y., Kantoff, P. W., et al. Modification of BRCA1- and BRCA2-associated breast cancer risk by AIB1 genotype and reproductive history. Cancer Res 2001; 61*:* 5420-4.
